# Segmented Echo Planar Imaging Improves Detection of Subcortical Functional Connectivity Networks in the Rat Brain

**DOI:** 10.1038/s41598-018-37863-2

**Published:** 2019-02-04

**Authors:** Stefano Tambalo, Giulia Scuppa, Angelo Bifone

**Affiliations:** 1 Center for Neuroscience and Cognitive Systems, Istituto Italiano di Tecnologia, Corso Bettini 31, 38068 Rovereto, Italy; 2 Department of Molecular Biotechnologies and Health Sciences, University of Torino, Via Nizza 52, 10126 Torino, Italy

## Abstract

Susceptibility artifacts in the vicinity of aural and nasal cavities result in significant signal drop-out and image distortion in echo planar imaging of the rat brain. These effects may limit the study of resting state functional connectivity in deep brain regions. Here, we explore the use of segmented EPI for resting state fMRI studies in the rat, and assess the relative merits of this method compared to single shot EPI. Sequences were evaluated in terms of signal-to-noise ratio, geometric distortions, data driven detection of resting state networks and group level correlations of time series. Multishot imaging provided improved SNR, temporal SNR and reduced geometric distortion in deep areas, while maintaining acceptable overall image quality in cortical regions. Resting state networks identified by independent component analysis were consistent across methods, but multishot EPI provided a more robust and accurate delineation of connectivity patterns involving deep regions typically affected by susceptibility artifacts. Importantly, segmented EPI showed reduced between-subject variability and stronger statistical significance of pairwise correlations at group level over the whole brain and in particular in subcortical regions. Multishot EPI may represent a valid alternative to snapshot methods in functional connectivity studies, particularly for the investigation of subcortical regions and deep gray matter nuclei.

## Introduction

The use of resting-state fMRI in translational research is a powerful tool to investigate the effects of aberrant brain connectivity in animal models of neurological and psychiatric disorders. The most exploited contrast in fMRI reflects physiological changes in blood oxygenation levels that ultimately lead to fluctuations in the T2*-weighted MR signal. This effect, known as BOLD (Blood Oxygenation Level Dependent) contrast^1^, provides a surrogate marker of brain activity, as shown by a growing and well established body of literature^2^. Resting state functional MRI in rodents is challenging for a number of reasons, first and foremost for the relative size of the brain compared to that of humans. Higher magnetic fields are often the first choice among a number of solutions to improve SNR, spatial resolution and sensitivity to subtle fluctuations of the BOLD signal in the resting brain. However, there are some costs in terms of off-resonance effects and geometric distortions, artifacts directly related to the applied magnetic field strength. These drawbacks are particularly severe in EPI sequences with gradient-echo envelope, which are the most popular pulse sequences in functional MRI and in resting state fMRI in particular. Off-resonance artifacts arise from B0 inhomogeneity, and are most prominent where the magnetic susceptibility mismatch between tissues is large. In the vicinity of air-tissue interfaces, the magnitude of signal dropouts and geometric distortions can be sufficiently high to corrupt anatomical information or functional measurements^3^. This effect is particularly pronounced in brain regions adjacent to acoustic and olfactory external meatus and esophagus. Deep gray matter nuclei, amygdala and hypothalamus are some of the areas negatively affected by these artifacts. Geometric distortions caused by off-resonance effects are much more evident in the phase encoding (PE) direction, as frequency shifts accumulated along the readout direction are negligible compared to the high bandwidth per pixel. In the PE direction the bandwidth is much lower, and even small errors in phase encoding result in significant stretching or shrinking of the image. Several solutions have been proposed to compensate for phase errors distortions. Image dewarping is a fairly common strategy that requires a B0 map obtained from the phase difference of two images acquired at different echo times. Each voxel in the map is an estimation of the phase - and thus position - shift caused by field inhomogeneity. The corrected image is then obtained by inverting these shifts and resampling the functional data in a regularly spaced grid. This method greatly improves coregistration to structural or standard spaces, as long as overall movement is limited and B0 maps are sufficiently regularized through appropriate spatial smoothing^4,5^. Other solutions could be applied to the pulse sequence. Faster readouts, higher bandwidths, increased gradients strengths and shorter echo spacing, all lead to faster k-space filling, leaving less time for phase errors to accumulate. These fine adjustments of the pulse program are constrained by hardware limitations and, especially for human studies, by fast gradient field switching that in some cases could lead to peripheral nerve stimulation. On the hardware side, parallel acquisition could also be a viable solution. Shorter readout times are obtained by skipping k-space lines, and missing data are inferred during image reconstruction. The use of multi-channel array coils is mandatory for an efficient application of this method and acceleration factors of 2–4 appear to be achievable with satisfactory results even at high field strengths^6,7^. If controlling distortion is a priority, in addition to fine tuning of the sequence and post processing, one may also consider to use pulse sequences that are inherently less susceptible to distortion. Multishot EPI falls in this class of imaging sequences. The strategy of partitioning the k-space is referred to as segmentation; in multishot EPI each k-space segment is acquired after a radiofrequency pulse, or “shot”, and images are obtained from the reconstructed k-space. A major advantage of this approach is the shortening of the readout time. The net result of using multiple excitations is that off-resonance artifacts and phase errors are effectively reset on each RF pulse, with tangible improvements in spatial resolution and reduction of geometric distortions compared to conventional single shot EPI^8^. On the other hand, significant disadvantages are represented by reduction in frame rate and increased sensitivity to inter-shot instabilities^9^ that may arise from physiological sources of motion or instrumental effects. Nevertheless, segmented EPI has been proven beneficial in certain functional MRI applications^10–12^. To date, to the best of our knowledge, the pros and cons of multishot EPI for functional connectivity studies have not been thoroughly investigated. Here we apply a robust and minimally invasive protocol to assess the relative benefits of single- and multishot EPI for resting state fMRI in the rat brain at 7 Tesla, with particular attention to subcortical and ventral regions, where susceptibility artifacts are most prominent. These circuits play a crucial role in many psychiatric disorders, as they are involved in the processing of goal-directed behavior, movement control, cognition and emotion. Therefore their accurate detection is important to monitor the modulation of functional connectivity in translational models of neuropsychiatric diseases. In this work, we propose segmented EPI as a viable alternative to conventional echo planar imaging as it shows improved SNR, temporal SNR and a more robust and accurate delineation of deep brain regions, whose detection is normally negatively affected by B0 inhomogeneity.

## Results

### Image quality assessment

**Figure 1:**
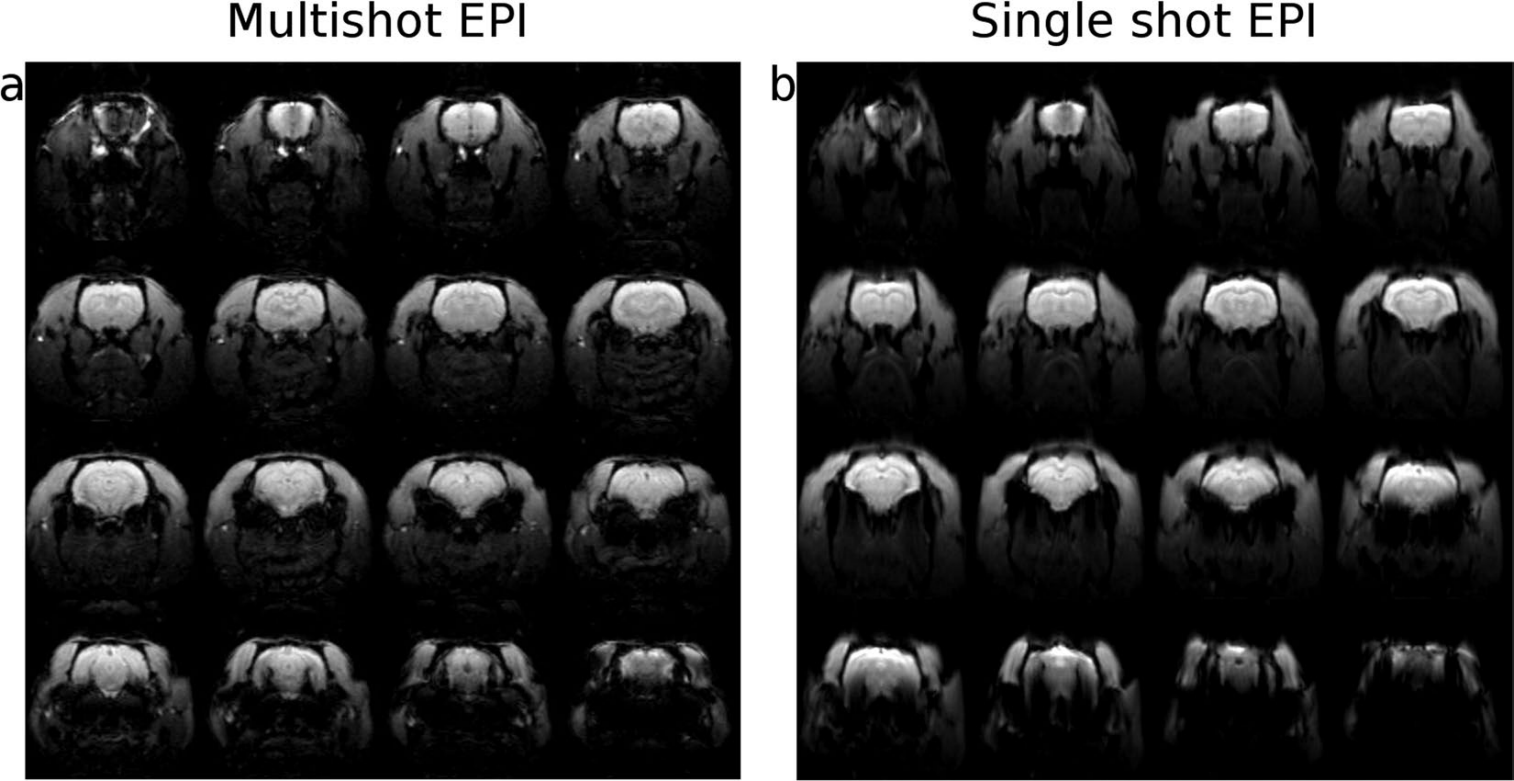
Echo-Planar Imaging comparison. Representative functional datasets obtained with (**a**) and without (**b**) segmentation of the k-space in EPI pulse sequence. Image distortion and susceptibility artifacts are reduced in multishot imaging.

**Figure 2:**
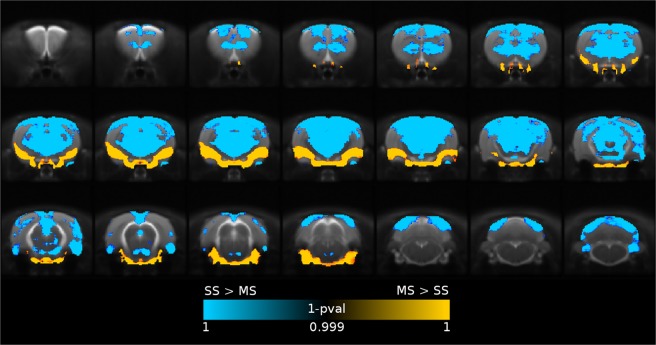
Spatial distribution of tSNR. MS-EPI shows reduced temporal SNR in cortical regions (blue), while improving the overall quality of time series in Amygdala, Insular Cortex, Olfactory Nuclei, Olfactory Tubercle, and Hypothalamic subregions (yellow).

Representative images obtained with the two sequences are reported in Fig. 1 (panel a: segmented EPI, panel b: conventional EPI). Geometric distortions and susceptibility artifacts are more severe in the conventional case. As expected, multishot EPI improves the SNR due to shorter readout time that reduces susceptibility distortions and blurring. The quality of the associated time series in susceptibility affected regions, like the Amygdala (p = 0.021) is therefore improved (SNR comparison is reported in Fig. S1, Supplementary Information). In some cortical ROIs, SNR is reduced by a factor of 2 when compared to single shot EPI. Despite this, the values recorded are higher than the minimum required SNR to detect a 1% BOLD signal change^13^. The temporal SNR was tested voxelwise with nonparametric methods to assess statistically significant differences. Figure 2 shows the spatial distribution of tSNR variation between the sequences. While the general tSNR is higher in SS-EPI, improvements are obtained in subcortical areas with MS-EPI. In particular, Amygdala (MS-EPI: 56.8 ± 5.2, SS-EPI: 40.1 ± 1.8, p < 0.05), Insular Cortex (MS-EPI: 134.7 ± 6.3, SS-EPI: 118.8 ± 8.3, p < 0.05), Olfactory Nuclei (MS-EPI: 79.8 ± 1.3, SS-EPI: 61.5 ± 2.1, p < 0.05) and Olfactory Tubercle (MS-EPI: 67.8 ± 0.6, SS-EPI: 57.6 ± 0.9, p < 0.05), all benefit from a substantial recovery of temporal SNR with segmentation of k-space in echo planar imaging. Concerning the impact of motion, both sequences showed no significant difference (p > 0.4) in the number of outlier volumes per subject, with an average of 1% of discarded time points in SS-EPI and 3% in the case of MS-EPI.

### Geometric distortion

**Figure 3:**
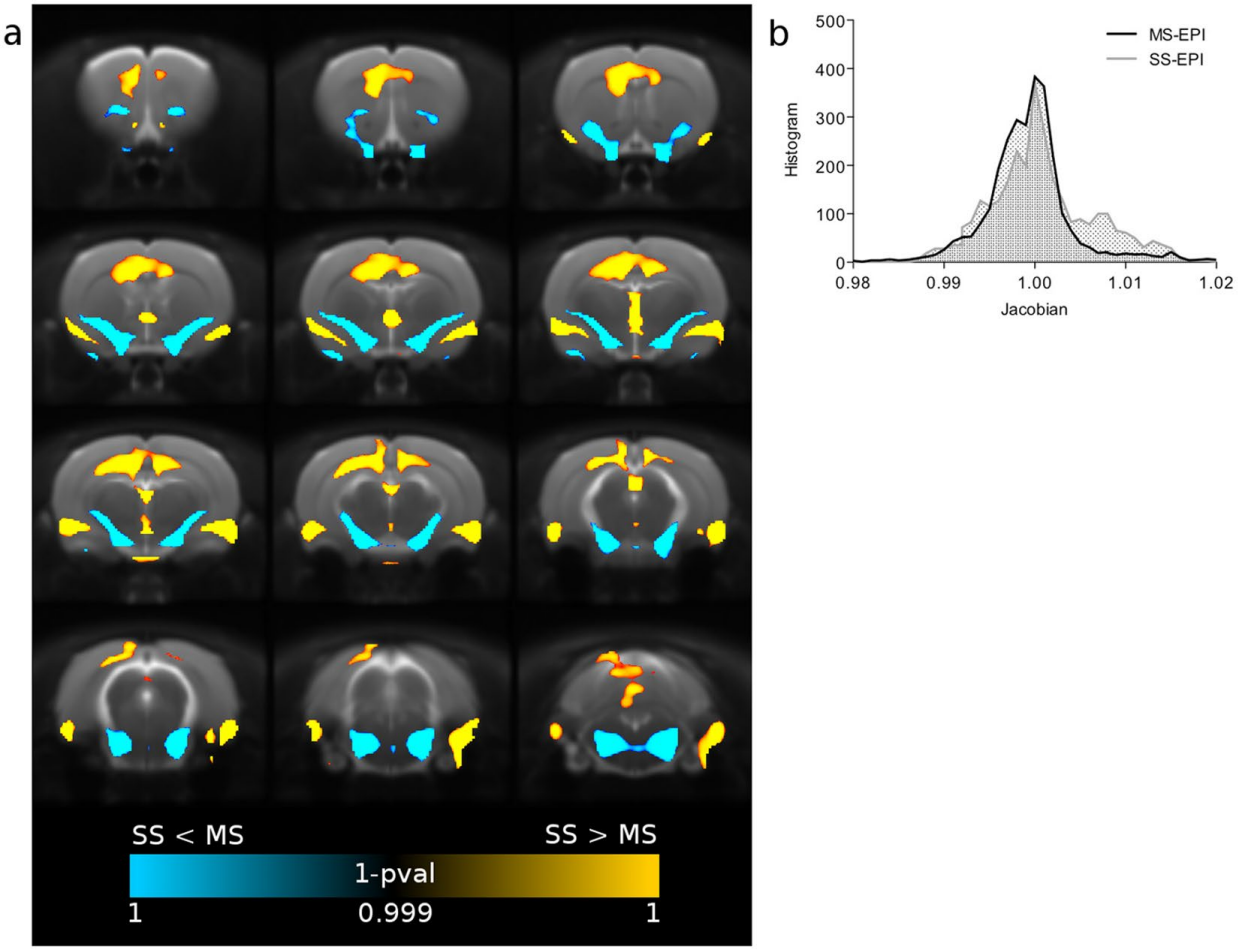
Geometric distortion of EPI sequences. Panel a: voxelwise (TFCE, FWE-corrected) parametric map of local deformations corrected by nonlinear registration. SS-EPI introduces severe image distortions compared to MS-EPI in terms of local expansion (yellow) or compression (blue). Panel b: distribution of Jacobian coefficient.

Multishot echo planar imaging improved the accuracy of nonlinear registration to standard space rat brain template (Dice index, MS-EPI: 0.86 ± 0.02, SS-EPI: 0.84 ± 0.02, p < 0.01). In addition to the similarity index between warped EPI scans and rat brain template, we considered also the Jacobian determinant, a relative measure of the local expansions and compressions applied to the original image (in native space) to obtain the warped image (normalized to template space). Figure 3, panel a, shows the voxelwise parametric map of local deformations corrected by nonlinear registration in SS-EPI compared to MS-EPI. To match the native space of functional images to the template space, subcortical and medial regions of the brain require significant expansions, while higher compression of the volume is required in the thalamic areas, compared to MS-EPI. Panel b shows the histogram of Jacobian coefficients. As expected, a heavy tail towards values greater than 1 can be noticed in the SS-EPI distribution.

### Independent component analysis

**Figure 4:**
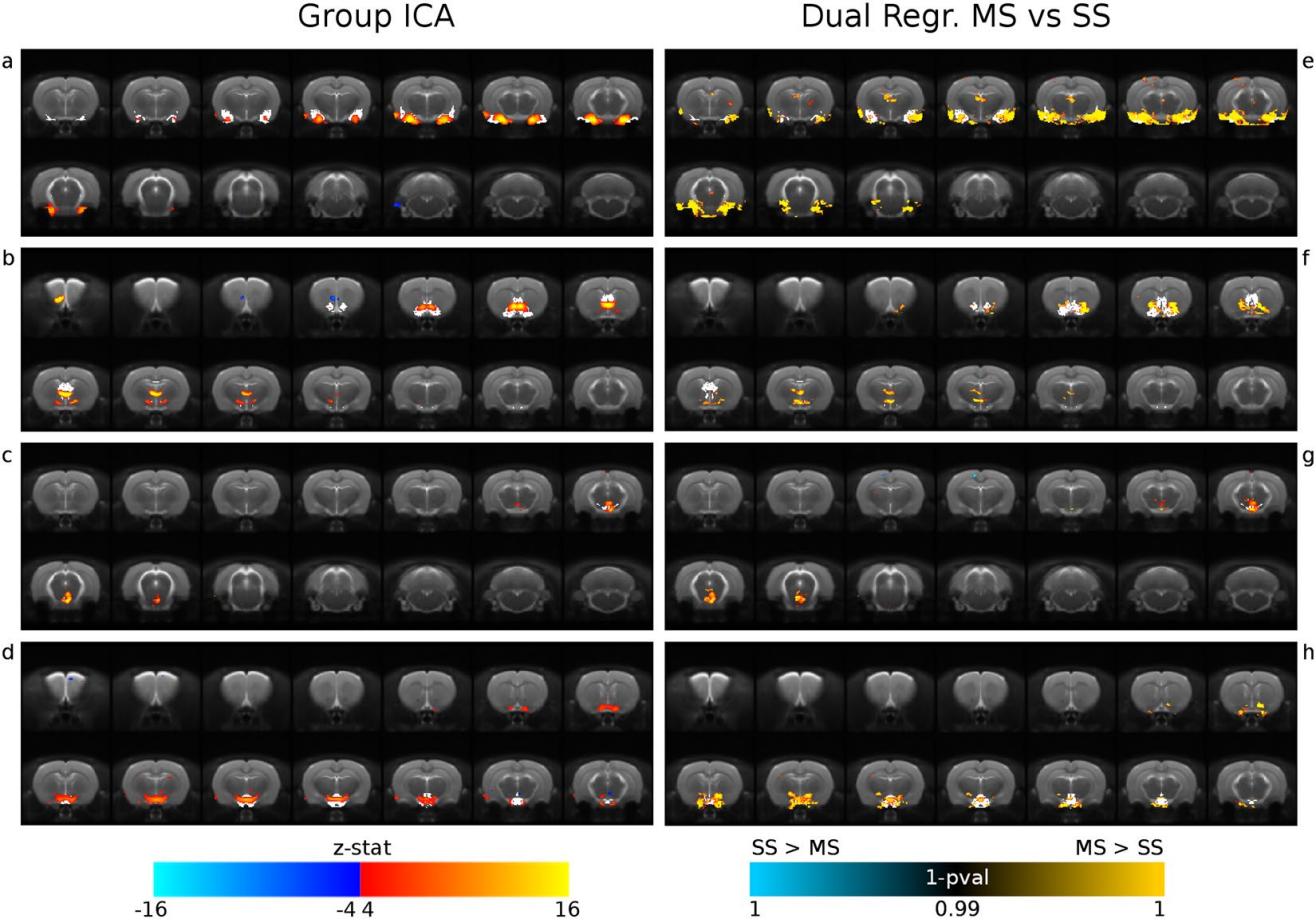
Independent Component Analysis and Dual Regression. Group specific differences in subcortical resting state networks (panel a–d: Amygdala, Nucleus Accumbens/Septum, VTA, Hypothalamus) are investigated with a dual regression approach. MS-EPI provides significantly improved delineation of subcortical components over SS-EPI. Corresponding anatomical regions, extracted from the rat brain atlas and used as reference, are superimposed in white.

Resting state networks returned by group-level ICA were labelled as: Salience, Sensory, Posterior Sensorimotor, Posterior Cingulate, Anterior Cingulate, Mesolimbic System, Amygdala, Nucleus Accumbens/Septum, VTA and Hypothalamus. A number of RSNs (See Fig. S2, Supplementary Information; red and blue color scales refer to MS-EPI and SS-EPI, respectively) show a good degree of overlap and consistency between cortical components obtained with segmented and SS-EPI. Improvement in delineation of connectivity patterns by MS-EPI is observed in deeper brain structures, e.g. in the Salience Network, where the spatial component shows a better anatomical correspondence with the Insular cortex. Evaluation of sequence-specific differences in the detection of functional networks was implemented with a dual regression analysis, with special attention to subcortical components. Results are reported in Fig. 4. Significant differences between MS and SS EPI in functional connectivity at group level were detected in the Amygdala, Hypothalamus, VTA and the Nucleus Accumbens/Septum components (TFCE, FWE-corrected, 1-p > 0.99). Particularly, MS-EPI shows significantly improved delineation of these brain regions when compared to the SS-EPI (Fig. 4, panels e–g).

### Consistency of pairwise correlations

**Figure 5:**
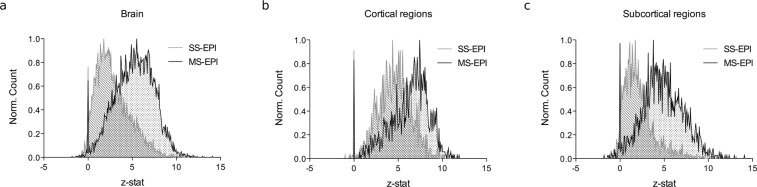
Consistency of pairwise correlations. Distribution of pairwise z-stat at the group level between MS-EPI and SS-EPI over the whole brain (**a**), in cortical (**b**) and in subcortical areas (**c**). MS-EPI globally improves the estimation of correlation coefficients at the group level (p < 0.01), especially in subcortical regions (p < 0.001).

Distributions of one-sample z-stats of adjacency matrices across subjects for both acquisition methods are reported in Fig. 5. MS-EPI shows higher z-stat values (p < 0.01) and a right-shifted distribution, features that indicate increased statistical power and reduced sample size required for group-level analysis of pairwise temporal correlations. The major advantage of MS-EPI emerges in deep gray matter nuclei (e.g. Nucleus Accumbens, Amygdala, Thalamus, Hypothalamus, Raphe, Ventral Tegmental Area), where a statistically significant difference in the distribution of z-stats is observed (p < 0.001). Considering z = 2.3 as the cluster forming threshold to assess the validity of pairwise correlations, and given a desired power level (1-β) of 0.8, a power analysis indicates that the sample size required to detect significant correlations in the aforementioned regions, with α = 0.05, would be n = 11 for MS-EPI and n = 31 for SS-EPI. These differences are amplified for more conservative values of the threshold. On the other hand, no differences are reported for cortical regions. When considering z-score of the correlation coefficient at a group level over the whole brain, the ratio of average z-score to its standard deviation is larger for MS-EPI compared to SS-EPI (MS-EPI: 1.39, SS-EPI: 1.02). This ratio is comparable for cortical regions (MS-EPI: 1.65, SS-EPI: 1.54), but significant divergence was observed for brain regions usually affected by poor SNR (MS-EPI: 1.23, SS-EPI: 0.84).

## Discussion and Conclusion

Echo Planar Imaging is the gold standard pulse sequence in resting state connectivity studies as it conjugates excellent sensitivity to T2* variations over time to high temporal resolution^14,15^. These factors are equally critical when measuring spontaneous functional activity of the brain. On the other hand, greater sensitivity to susceptibility mismatch is also the source of planar and temporal artifacts that could reduce the signal to noise ratio of the functional time series, particularly in brain regions close to the auditory channels and nasal meatus. Here we compared a conventional snapshot EPI sequence with a multishot version that exploits k-space segmentation to reduce the sensitivity to artifacts. Preclinical applications of functional magnetic resonance imaging are the result of a well-balanced combinations of experimental, technical and ethical constraints. Regarding the latter, even under the principles of justification and optimization, and given the intrinsic non-invasive nature of MRI, it is often necessary to trade protocol invasiveness for superior image quality, which ultimately translates in reliable outcomes. Several promising methods, such as filling ear cavities with toothpaste or fluorocarbon, have been proposed^3,16^ with remarkable results. Although the gain in image quality is beyond dispute, similar methods face some limitations in terms of invasiveness and applicability, e.g. in the case of longitudinal or within-subject experimental designs. On the technical side, parallel imaging methods^17,18^ are at least as effective as interleaved EPI in terms of signal restoration and reduction of image warping. However, the RF coils designed for the rat brain and the limited number of receiver channels available (compared to their human counterpart) do not allow to take full advantage of parallelization methods. Based on these considerations, when experimental procedures include a longitudinal design and/or minimization of invasiveness is a major constraint, interleaved echo-planar acquisitions represent a viable option. Multishot imaging provides improved SNR and tSNR in brain areas heavily affected by intrinsic B0 inhomogeneity, while maintaining sufficiently high SNR over the whole brain. Geometric distortion is also greatly reduced in the same areas, with substantial advantages in anatomical localization and reduction of potential artifacts introduced by spatial normalization.

Resting state networks identified by independent component analysis are reliable and consistent across methods, at least in cortical regions. However, it is important to note that substantial improvements in the detection of functional networks are introduced by multishot EPI when looking at subcortical regions, probably as a consequence of the increased SNR and tSNR and the effective control of geometric distortion in these areas.

Finally, consistency of correlation statistics between subjects is enhanced in the multi-shot EPI dataset compared to the single-shot EPI. It is interesting to note the differential magnitude of the effect in cortical and subcortical areas. This feature suggests a significant gain in sensitivity in specific regions of the brain that are particularly affected by susceptibility artifacts. This is critically important for group-level resting state FC studies as it improves statistical power, thus reducing the number of subjects required to reach statistical significance, in accordance with the RRR principles^19^. Segmented EPI may thus represent a valid alternative to snapshot methods in functional connectivity studies, especially when subcortical regions and deep gray matter nuclei are critical targets.

## Materials and Methods

Images were acquired at 7 T on a Bruker Pharmascan (https://www.bruker.com) in a double coil configuration. A 72 mm i.d. single channel transmission-only resonator was actively decoupled with a four channel phased-array receive-only surface coil optimized for the rat brain. Pulse sequence parameters for multishot gradient echo (MS-EPI) acquisition were set as follows: TR = 500(x4)ms, TE = 17 ms, BW = 250 kHz, NSHOTS = 4, NEX = 1, MTX = 96 × 96 × 16, FOV = 35 × 35 × 16 mm, NREP = 900, PPI = 2, ACQtime = 30 min. The same RX/TX configuration was used for conventional single shot gradient echo EPI (SS-EPI), and parameters were adjusted as follows: TR = 1000 ms, TE = 17 ms, BW = 250 kHz, NSHOTS = 1, NEX = 1, MTX = 96 × 96 × 16, FOV = 35 × 35 × 16 mm, NREP = 1800, PPI = 2, ACQtime = 30 min. We studied the global SNR as a function of flip angles ranging from 30 to 90°. A flip angle of 60° was selected as it gives near-optimal, comparable levels of SNR for both sequences over the whole brain (see Supplementary Information, Fig. S3). The contribution of inflow effect in modulating the BOLD signal at this field strength and with relatively long TRs is negligible^20^, so inflow compensation was not implemented. Navigator echo was used in both sequences and global shimming was refined by localized shimming with FASTMAP protocol. In addition, high resolution T2w RARE images (TR = 5500 ms, TE = 76 ms, NEX = 8, MTX = 256 × 256 × 25, FOV = 35 × 35 × 25 mm, ACQtime = 5 min 20 s) were acquired for anatomical reference and to drive the co-registration step of the preprocessing.

### Animal preparation

Two distinct groups of Wistar rats (Envigo, Italy) weighing around 300 gr were used (MS-EPI: n = 27, SS-EPI: n = 25). Animals were housed in pairs under controlled environmental conditions with free access to water and food pellets. For the rs-fMRI experiment, we adopted a minimally invasive protocol, as described in^21^ and adapted from^22,23^. Animals were initially anesthetized with 3% isoflurane (Vetflurane, VIRBAC SA, Carros, France) in O_2_:N_2_O (3:7), which was then reduced to 1.5% for maintenance during preparation^24^. Subsequently, a bolus of 0.05 mg/kg/ml of medetomidine (Domitor, 1 mg/ml, Orion Corporation, Espoo, Finland) was injected intraperitoneally, followed, 15 minutes later, by a continuous infusion of medetomidine diluted 1:4 in saline at a rate of 1 ml/kg/h through a catheter inserted subcutaneously. At this point, isoflurane was discontinued. During the scan session, respiration rate, heart rate and blood oxygen saturation were monitored with a pulsoximeter (Starr Life Sciences Corp. Oakmont, PA). CO_2_ concentration in the blood was also assessed using a TCM4 Transcutaneous Blood Gas Analyzer (Radiometer Medical ApS, Denmark). Rectal temperature was maintained at 37 °C during the experiments by a feedback-controlled, water-circulating heating pad. At the end of the fMRI experiment, medetomidine was antagonized by an intraperitoneal injection of atipamezole (Antisedan, 0.1 mg/kg; Orion Corporation, Espoo, Finland). The experimental protocol received authorization from the Italian Ministry of Health (approval numbers 796/2016-PR) and was approved by the local ethical committee. Animal work was conducted following Italian law (DL 1992 and subsequent modifications) and the European Union (2010/63/EU) directive.

### Image analysis

Images were processed with MATLAB (MathWorks, Natick, MA) and FSL (http://fmrib.ox.ac.uk/fsl). Raw data were converted to nifti format and voxel size was scaled by a factor of 10 to be compliant with FSL algorithms. Preprocessing consisted of motion correction, estimation and regression of 12 motion confounds (translation/rotation and their derivatives), regression of volumes corrupted by excessive motion, linear detrending, slice timing correction, nonlinear filtering of image noise, nonlinear high-pass and gaussian low-pass spectral filtering of time series (0.01–0.1 Hz). Functional images were coregistered by 12 DOF affine transformations to structural T2w anatomical references and then resampled to a rat brain template in standard space^25^. After rigid body transformation, nonlinear warping to standard space was applied to further refine the normalization and minimize geometric distortions.

### Image quality assessment

SNR and tSNR were calculated for both EPI methods with a ROI analysis approach to compare the overall quality of the measured time-series. We considered mean signal intensity of ROIs placed respectively in the Sensory Cortex S1, Caudate Putamen and Amygdala, while a reference ROI was placed in the background to obtain standard deviation of the noise level. A two sample t-test was then applied to compare SNR at the subject level for the two acquisition methods. Temporal SNR is often employed as a measure of sensitivity of a pulse sequence, and was calculated voxelwise as the mean intensity of the time course divided by its standard deviation. Differences in tSNR were assessed by non-parametric permutation test and reported as TFCE maps of 1 – p value, FWE-corrected. Improvement in susceptibility-induced geometrical distortions of the multishot EPI were derived by computing the Dice similarity index at the subject level with the standard space rat brain template and compared with an unpaired t-test. Moreover, as for tSNR measurements, voxelwise values of the jacobian determinant of warp fields obtained by nonlinear spatial normalization were analyzed by non-parametric permutation test and reported as TFCE maps of 1 – pvalue, FWE-corrected. We also investigated the impact of motion artifacts on the two sequences, given the higher sensitivity of MS-EPI to subtle movements, ghosting and shot-to-shot instabilities. DVARS was used to compute the number of outlier volumes for every subject in the two groups. Values were tested for sequence-dependent differences with a two sample t-test.

### Resting state networks identification

Independent component analysis with a high number of components (n = 70) was run at the subject level. ICA components were then hand-classified in a randomly sampled subset of subjects and labeled as noise or signal on the basis of spatial maps, frequency content and time-course. This approach was effective in separating components related to subtle whole-head motion, CSF flow, ghosting and between-shots motion artifacts in the segmented EPI. Spatial maps non related to anatomical structures, characterized by shifts towards high frequencies in the power spectrum or corrupted by clearly visible spikes were classified as noise. Labels were used to train an automated classifier and non-aggressive regression of noisy components was applied to the whole set of functional images^26,27^. The accuracy of the classifier was assessed using a leave-one-out test and scored a true positive rate (proportion of “good” components correctly labeled) higher than 95%. Denoised functional data were finally used to extract group level ICA with a reduced number of components (d = 30) to obtain data-driven resting state networks (RSNs) on a single joint dataset that included both EPI acquisition methods, to obtain an unbiased estimation of the group-level components. Components localized in subcortical regions (6 out of 30 components) were considered for statistical comparison with a dual regression approach, to test whether MS-EPI could improve the detection of resting state networks connectivity in these regions, as reported in^28^.

### Consistency of pairwise correlations

Reliability of adjacency matrices obtained by both sequences was also examined. Time series corresponding to 70 brain regions for each hemisphere were extracted by atlas-based anatomical parcellation of functional images^29^. To rule out any spurious effect related to the different number of time points acquired for the two sequences, SS-EPI data were resampled to match the sampling frequency of MS-EPI data. Pearson’s R was then used to measure temporal correlation of BOLD signal fluctuations at subject level. Fisher-transforms of correlation coefficients in the adjacency matrices were calculated for every subject and tested with one-sample t-test for each acquisition method. Robustness of pairwise correlations at group level was defined as the z-statistic of the t-test.

The datasets generated and analyzed during the current study are available from the corresponding author on reasonable request.

## Supplementary information

Supplementary Information

## References

[1] Ogawa S, Lee TM, Kay AR, Tank DW (1990). Brain magnetic resonance imaging with contrast dependent on blood oxygenation. Proc. Natl. Acad. Sci. USA.

[2] Attwell D, Iadecola C (2002). The neural basis of functional brain imaging signals. Trends Neurosci..

[3] Mandeville JB (1998). Dynamic functional imaging of relative cerebral blood volume during rat forepaw stimulation. Magn. Reson. Med..

[4] Cusack R, Brett M, Osswald K (2003). An evaluation of the use of magnetic field maps to undistort echo-planar images. Neuroimage.

[5] Hutton C (2002). Image distortion correction in fMRI: A quantitative evaluation. Neuroimage.

[6] Preibisch C (2003). Functional MRI using sensitivity-encoded echo planar imaging (SENSE-EPI). Neuroimage.

[7] Speck O, Stadler J, Zaitsev M (2008). High resolution single-shot EPI at 7T. Magn. Reson. Mater. Physics, Biol. Med..

[8] Lu H, Mazaheri Y, Zhang R, Jesmanowicz A, Hyde JS (2003). Multishot partial-k-space EPI for high-resolution fMRI demonstrated in a rat whisker barrel stimulation model at 3t. Magn. Reson. Med..

[9] Menon, R. S., Thomas, C. G. & Gati, J. S. Investigation of BOLD contrast in fMRI using multi-shot EPI. *NMR Biomed*. **10**, 179–82 (1997). 10.1002/(sici)1099-1492(199706/08)10:4/5<179::aid-nbm463>3.0.co;2-x 9430345

[10] Hoogenraad FG (2000). High-resolution segmented EPI in a motor task fMRI study. Magn. Reson. Imaging.

[11] Guilfoyle DN, Hrabe J (2006). Interleaved snapshot echo planar imaging of mouse brain at 7.0 T. NMR Biomed..

[12] Swisher JD, Sexton JA, Gatenby JC, Gore JC, Tong F (2012). Multishot versus single-shot pulse sequences in very high field fMRI: a comparison using retinotopic mapping. PLoS One.

[13] Parrish TB, Gitelman DR, LaBar KS, Mesulam MM (2000). Impact of signal-to-noise on functional MRI. Magn. Reson. Med..

[14] Biswal B, Yetkin FZ, Haughton VM, Hyde JS (1995). Functional connectivity in the motor cortex of resting human brain using echo-planar MRI. Magn. Reson. Med..

[15] Howseman AM, Thomas DL, Pell GS, Williams SR, Ordidge RJ (1999). Rapid T2* mapping using interleaved echo planar imaging. Magn. Reson. Med..

[16] Li R (2015). Restoring Susceptibility Induced MRI Signal Loss in Rat Brain at 9.4 T: A Step towards Whole Brain Functional Connectivity Imaging. PLoS One.

[17] Sodickson DK, Manning WJ (1997). Simultaneous acquisition of spatial harmonics (SMASH): fast imaging with radiofrequency coil arrays. Magn. Reson. Med..

[18] Pruessmann KP, Weiger M, Scheidegger MB, Boesiger P (1999). SENSE: sensitivity encoding for fast MRI. Magn. Reson. Med..

[19] Kilkenny C, Browne WJ, Cuthill IC, Emerson M, Altman DG (2010). Improving bioscience research reporting: The ARRIVE guidelines for reporting animal research. J. Pharmacol. Pharmacother..

[20] Gao J-H, Liu H-L (2012). Inflow effects on functional MRI. Neuroimage.

[21] Tambalo S (2015). Functional Magnetic Resonance Imaging of Rats with Experimental Autoimmune Encephalomyelitis Reveals Brain Cortex Remodeling. J. Neurosci..

[22] Pawela CP (2009). A protocol for use of medetomidine anesthesia in rats for extended studies using task-induced BOLD contrast and resting-state functional connectivity. Neuroimage.

[23] D’Souza DV (2014). Preserved Modular Network Organization in the Sedated Rat Brain. PLoS One.

[24] Gozzi A, Schwarz A, Crestan V, Bifone A (2008). Drug–anaesthetic interaction in phMRI: the case of the psychotomimetic agent phencyclidine. Magn. Reson. Imaging.

[25] Schwarz AJ (2006). A stereotaxic MRI template set for the rat brain with tissue class distribution maps and co-registered anatomical atlas: application to pharmacological MRI. Neuroimage.

[26] Salimi-Khorshidi G (2014). Automatic denoising of functional MRI data: Combining independent component analysis and hierarchical fusion of classifiers. Neuroimage.

[27] Griffanti L (2014). ICA-based artefact removal and accelerated fMRI acquisition for improved resting state network imaging. Neuroimage.

[28] Nickerson LD, Smith SM, Öngür D, Beckmann CF (2017). Using Dual Regression to Investigate Network Shape and Amplitude in Functional Connectivity Analyses. Front. Neurosci..

[29] Schwarz AJ (2006). A stereotaxic MRI template set for the rat brain with tissue class distribution maps and co-registered anatomical atlas: Application to pharmacological MRI. Neuroimage.

