# Supplementary material for: Segmented Echo Planar Imaging Improves Detection of Subcortical Functional Connectivity Networks in the Rat Brain
Source: Sci Rep. 2019 Feb 4;9:1397. doi: 10.1038/s41598-018-37863-2 (PMC6362052; doi:10.1038/s41598-018-37863-2)
Supplement: Supplementary file 1 — Supplementary Information [file 41598_2018_37863_MOESM1_ESM.docx]

**Segmented Echo Planar Imaging Improves Detection of**

**Subcortical Functional Connectivity Networks in the Rat Brain**

Stefano Tambalo*^1^, Giulia Scuppa, PhD^1^, and Angelo Bifone, PhD^1^.

^1^Center for Neuroscience and Cognitive Systems, Istituto Italiano di Tecnologia

Corso Bettini, 31 I-38068 Rovereto, Italy

*Corresponding author: Stefano Tambalo, stefano.tambalo@iit.it

**SUPPLEMENTARY INFORMATION**

*Fig. S1 – Signal to Noise Ratio*


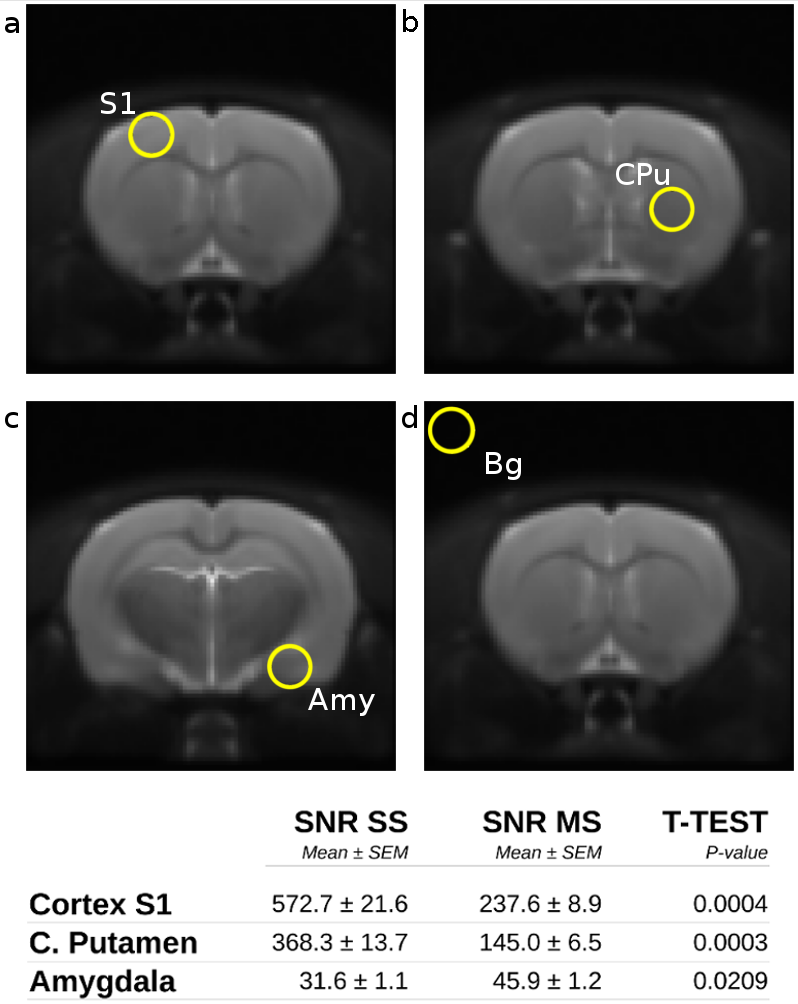


*Signal to Noise Ratio*. Regions of interest were placed in the Sensory Cortex S1 (a), Caudate Putamen (b), Amygdala (c) and background (d*)*. SNR is improved in the Amygdala (p=0.021) and reduced in Cortex S1 and Caudate Putamen in MS-EPI compared to SS-EPI.

*Fig. S2 – Independent Component Analysis*


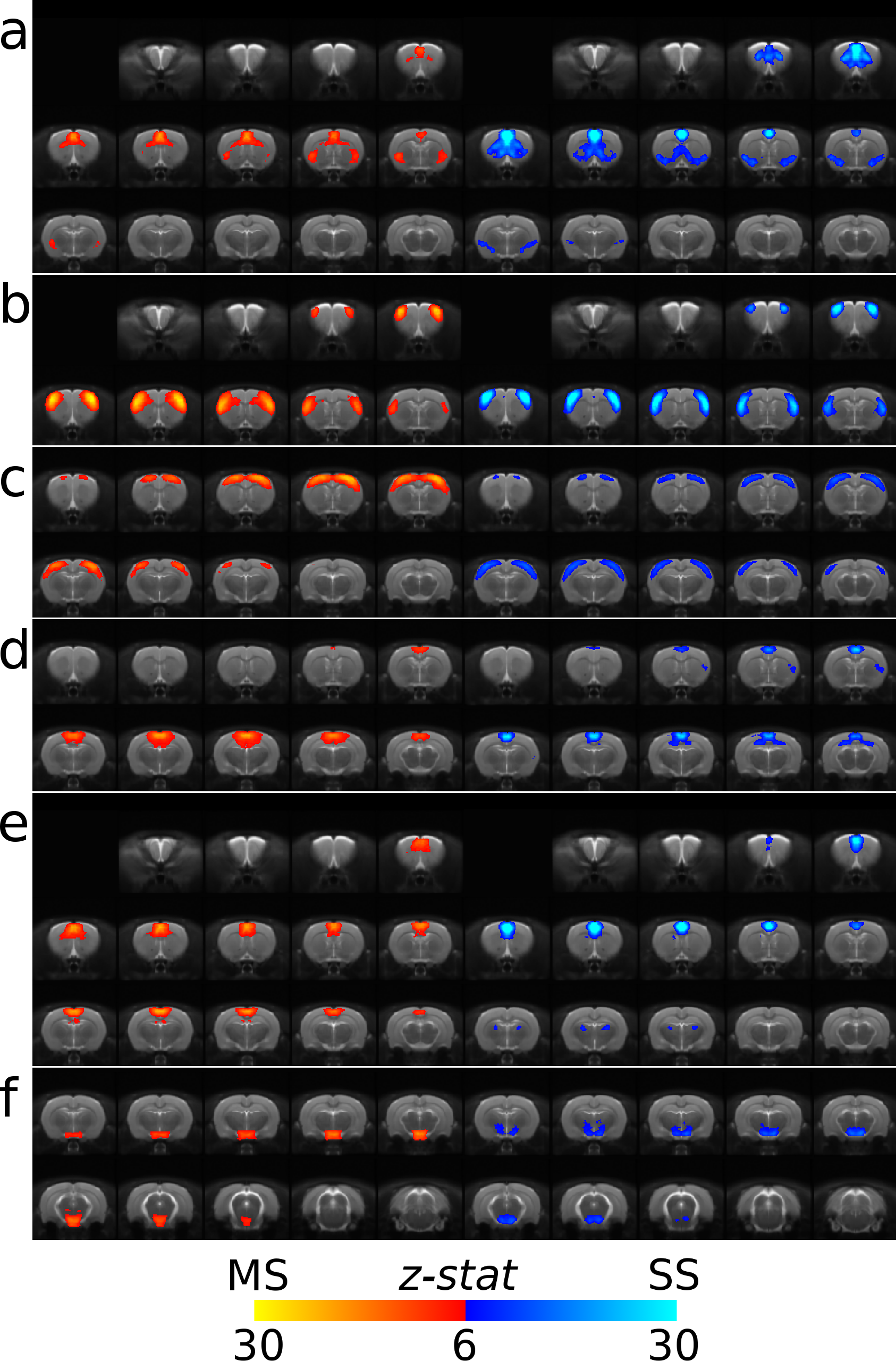


*Independent component analysis*. Comparable Resting State Networks identified by MS-EPI (red) and SS-EPI (blue): a) Salience Network, b) Sensory, c) Posterior Sensorimotor, d) Posterior Cingulate, e) Anterior Cingulate and f) Mesolimbic System.

*Fig. S3 – SNR as a function of Flip Angle*

*
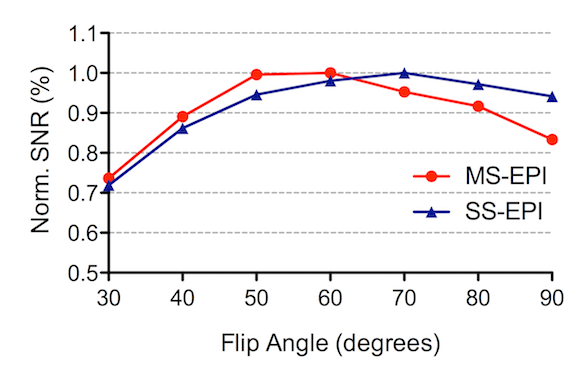
*

*SNR of MS-EPI and SS-EPI.* We studied the global SNR as a function of flip angles ranging from 30 to 90°. A flip angle of 60° gives near-optimal, comparable levels of SNR for both sequences over the whole brain.
